# Complex Hypertrophic Scar and Ventral Hernia Formation Following Laparoscopic Cholecystectomy: A Case Report

**DOI:** 10.7759/cureus.100646

**Published:** 2026-01-02

**Authors:** Shashawna S Drum Christie, Trevor Williams, Sabitra Niroula, Bom K Bong, Akwasi Berko, Ka Wai Wu, Frederick Tiesenga

**Affiliations:** 1 Medical School, St. George's University School of Medicine, St. George, GRD; 2 Medical School, St. George’s University School of Medicine, St. George, GRD; 3 Medical School, Rocky Vista University College of Medicine, Ivins, USA; 4 Medical School, Caribbean Medical University School of Medicine, Willemstad, CUW; 5 Medical School, Saint James School of Medicine, Arnos Vale, VCT; 6 Department of General Surgery, West Suburban Medical Center, Chicago, USA

**Keywords:** hypertrophic scar, incisional hernia, laparoscopic cholecystectomy, surgical site infection, triamcinolone injection

## Abstract

Hypertrophic scars are fibroproliferative lesions that form at the site of a wound due to excessive collagen deposition during the healing process. These scars remain confined to the original wound boundaries and may improve over time. Their formation is a complex process influenced by multiple factors, including wound depth and location, tension on the healing skin, genetic predisposition, and the inflammatory response. Abnormal regulation of fibroblasts and prolonged inflammation lead to excessive deposition of type III collagen (the final tensile collagen), resulting in a thickened, raised scar. Mechanical stress at the wound site, such as in areas of frequent movement (e.g., the abdomen), may also contribute to the development of exaggerated scarring. Additionally, delayed wound healing or infection, as seen in some postoperative cases, increases the risk of hypertrophic scar formation. Management often requires a multimodal approach involving pressure therapy, topical agents, corticosteroid injections, or surgical revision, depending on symptom severity. We present the case of a 46-year-old man with a complicated postoperative course who developed a symptomatic hypertrophic scar and a subsequent incisional hernia following laparoscopic cholecystectomy. Surgical excision of the hypertrophic scar with concurrent hernia repair and intralesional triamcinolone injection was performed; however, recurrent hypertrophic scarring developed postoperatively. Histopathologic examination confirmed the presence of dense collagen bundles, consistent with a hypertrophic scar. Although the patient achieved partial symptom relief with topical Contractubex^®^, he continued to experience tenderness and erythema at follow-up. This case outlines the complex interplay among infection, mechanical stress, and aberrant wound healing in the development of hypertrophic scars and incisional hernias. It further highlights the need for early recognition and a multimodal, individualized management strategy to optimize functional and cosmetic outcomes.

## Introduction

Hypertrophic scars represent abnormal wound-healing responses characterized by excessive collagen deposition, leading to raised, firm, and often symptomatic scars [1]. These lesions may develop after surgical trauma, postoperative infection, prolonged inflammation, or delayed wound healing and are often associated with pain, pruritus, and cosmetic concerns for patients. Hypertrophic scars occur in 39-68% of surgical wounds involving high-tension areas, with the abdomen representing a moderate-risk location due to frequent movement, increased dermal tension, and susceptibility to postoperative complications [1]. Postoperative complications, such as surgical site infection (SSI), have been shown to significantly increase the risk of abnormal scarring by disrupting normal granulation, impairing collagen organization, and prolonging inflammatory signaling [2]. Risk factors for poor wound healing include diabetes, obesity, stress, tobacco use, and malnutrition [2].

In addition to abnormal scar formation, impaired wound healing is a major risk factor for the development of incisional hernias, which occur in approximately 10-20% of abdominal surgeries, with even higher rates when infection is present. Mechanical tension, fascial dehiscence, and early collagen disarray weaken the integrity of the abdominal wall, thereby predisposing patients to herniation. Trocar-site hernias, although less common than midline incisional hernias, may occur at any laparoscopic port site, particularly those larger than 10 mm or those associated with postoperative infection or prolonged healing delay [3].

This case report describes the unusual presentation of a recurrent hypertrophic scar associated with an incisional hernia following laparoscopic cholecystectomy. Despite surgical revision, the patient continued to experience persistent pain, erythema, and thickening at the incision site, consistent with recurrent hypertrophic scarring. Partial symptomatic relief with topical Contractubex^®^ gel (heparin, allantoin, and onion extract) suggests that the irritation and tenderness were primarily related to ongoing abnormal scar tissue activity [4]. This case aims to highlight the interplay among postoperative infection, mechanical stress, and aberrant wound healing in the development of hypertrophic scars and incisional hernias following laparoscopic abdominal surgery.

## Case presentation

A 46-year-old man presented with persistent pain, swelling, and redness localized to a previous laparoscopic cholecystectomy incision site in the epigastric region. The patient underwent laparoscopic cholecystectomy on January 2, 2024, at a tertiary hospital for symptomatic acute calculous cholecystitis. The operation was challenging due to the presence of dense fibrotic tissue resulting from repeated inflammatory episodes. The gallbladder was removed laparoscopically after difficult dissection, during which bile spillage occurred, and the supraumbilical fascial incision required dilation to facilitate specimen extraction.

The immediate postoperative course was complicated by a superficial wound infection at the epigastric trocar site, manifesting as seropurulent drainage and localized tenderness. The infection was treated with oral cephalexin, which was discontinued due to an allergic rash. The wound subsequently healed by secondary intention. Although the infection resolved, the patient developed a progressively thickened, raised, erythematous scar that became tender and pruritic.

**Figure 1:**
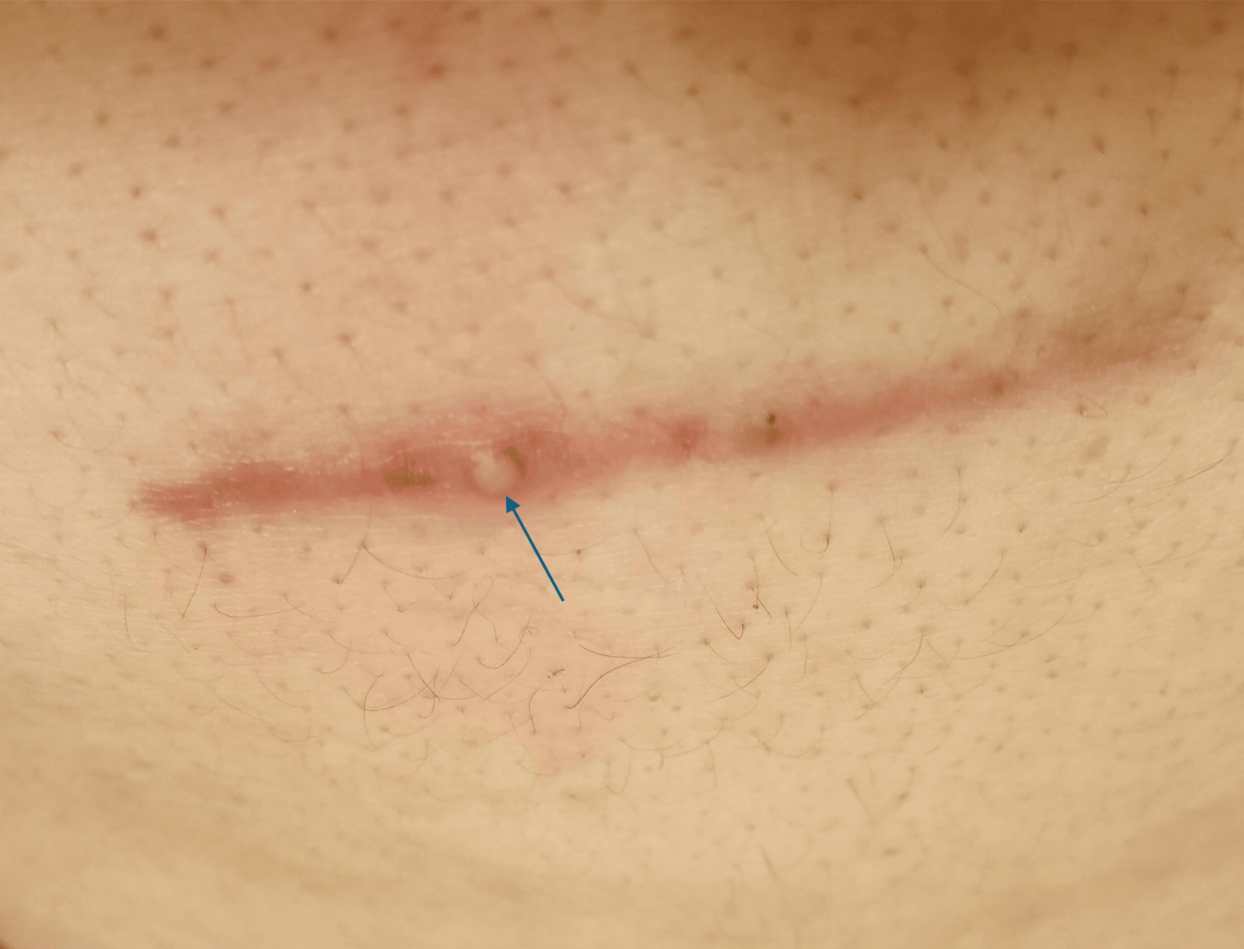
Clinical photograph of the patient’s postoperative epigastric incision site, demonstrating a linear, healed scar with focal areas of inflammation A central erythematous nodular elevation is visible along the scar line, consistent with localized inflammatory change. A small pustular focus is present just left of midline (blue arrow), representing superficial purulence within the scar tract. Surrounding the scar, there is mild diffuse erythema without induration or fluctuance. No active drainage is observed.

Over the following months, the patient noted progressive localized swelling and discomfort beneath the scar, described as a sensation of pressure and bulging (Figure 1). No inflammatory collection or mass was identified.

Persistent localized pain prompted further evaluation. Examination revealed a 1 × 1 cm incisional (ventral) hernia beneath a hypertrophic scar at the previous port site. Routine laboratory investigations demonstrated normal renal and hepatic function, normal albumin, and an elevated fasting glucose level consistent with prediabetes, which was managed non-pharmacologically. No recent HbA1c or inflammatory markers (CRP and ESR) were available. Laboratory findings are summarized in Table 1.

**Table 1:** Laboratory results at presentation

Test	Patient’s value	Reference range
Glucose (mg/dL)	106	70-99
Blood urea nitrogen (mg/dL)	16	7-25
Creatinine (mg/dL)	0.72	0.6-1.30
Sodium (mmol/L)	138	133-144
Potassium (mmol/L)	4.2	3.5-5.1
Chloride (mmol/L)	103	98-109
Carbon dioxide (mmol/L)	30	21-31
Anion gap (mmol/L)	5	3.6-11.0
Blood urea nitrogen/creatinine ratio	22	6.0-20.0
Calcium (mg/dL)	9.9	8.6-10.3
Total protein (g/dL)	7.3	6.4-8.9
Albumin (g/dL)	4.8	3.5-5.7
Aspartate aminotransferase (U/L)	11	13-39
Alanine aminotransferase (U/L)	21	7-52
Alkaline phosphatase (U/L)	64	40-129
Total bilirubin (mg/dL)	0.5	0.0-1.0
Glomerular filtration rate (Modification of Diet in Renal Disease, non-African American) (mL/min/1.73 m²)	>60	>60
Glomerular filtration rate (Modification of Diet in Renal Disease, African American) (mL/min/1.73 m²)	>60	>60

On February 13, 2025, the patient underwent open incisional hernia repair with excision of hypertrophic scar tissue and intralesional triamcinolone (Kenalog) injection at an outside hospital. Intraoperatively, a 1 × 1 cm fascial defect was identified beneath an area of dense fibrotic scar. The hernia sac and overlying hypertrophic scar were excised en bloc in an elliptical fashion. The fascial defect was closed with 0 Ethibond sutures, hemostasis was achieved, and the wound was closed in layers. Kenalog was injected into the wound bed to reduce the risk of recurrence. Estimated blood loss was minimal, and no complications occurred. A portion of the excised tissue was submitted for histopathologic evaluation.

**Figure 2:**
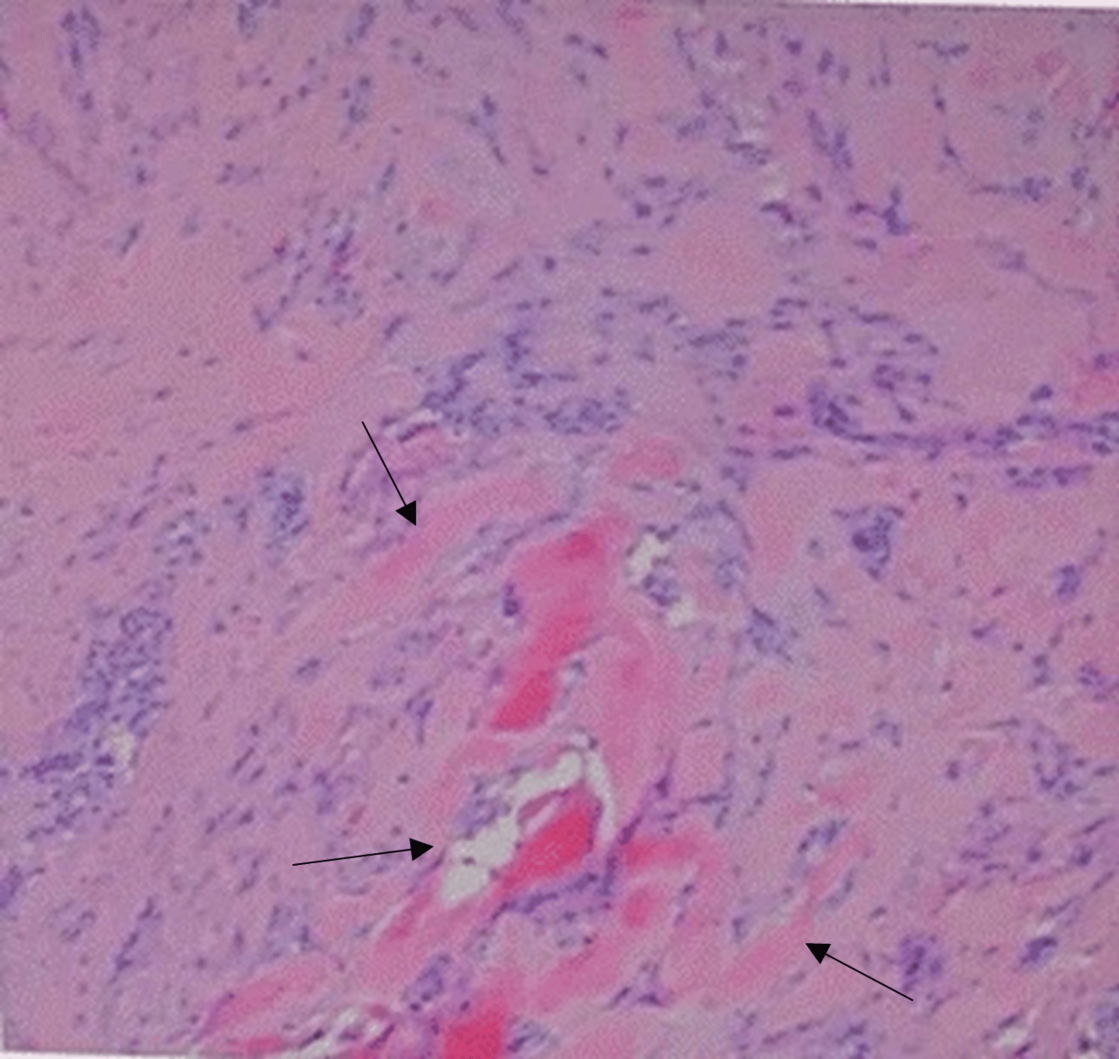
Histopathologic section of the excised hypertrophic scar Black arrows indicate dense eosinophilic collagen bundles within the dermis, accompanied by increased fibroblast proliferation (H&E stain, ×10). These findings are characteristic of hypertrophic scar architecture and confirm the absence of malignancy.

The pathology report (GoPath Laboratories; collected: February 13, 2025; reported: February 17, 2025) described a 3.3 × 1.1 × 1.0 cm segment of tan skin with underlying soft tissue and a second smaller lobulated fragment. Microscopic examination revealed dense, haphazardly arranged eosinophilic collagen bundles with fibroblastic proliferation, consistent with a hypertrophic scar. No atypia or malignancy was identified. The final pathology diagnosis was benign hypertrophic scar, without evidence of malignancy (Figure 2).

**Figure 3:**
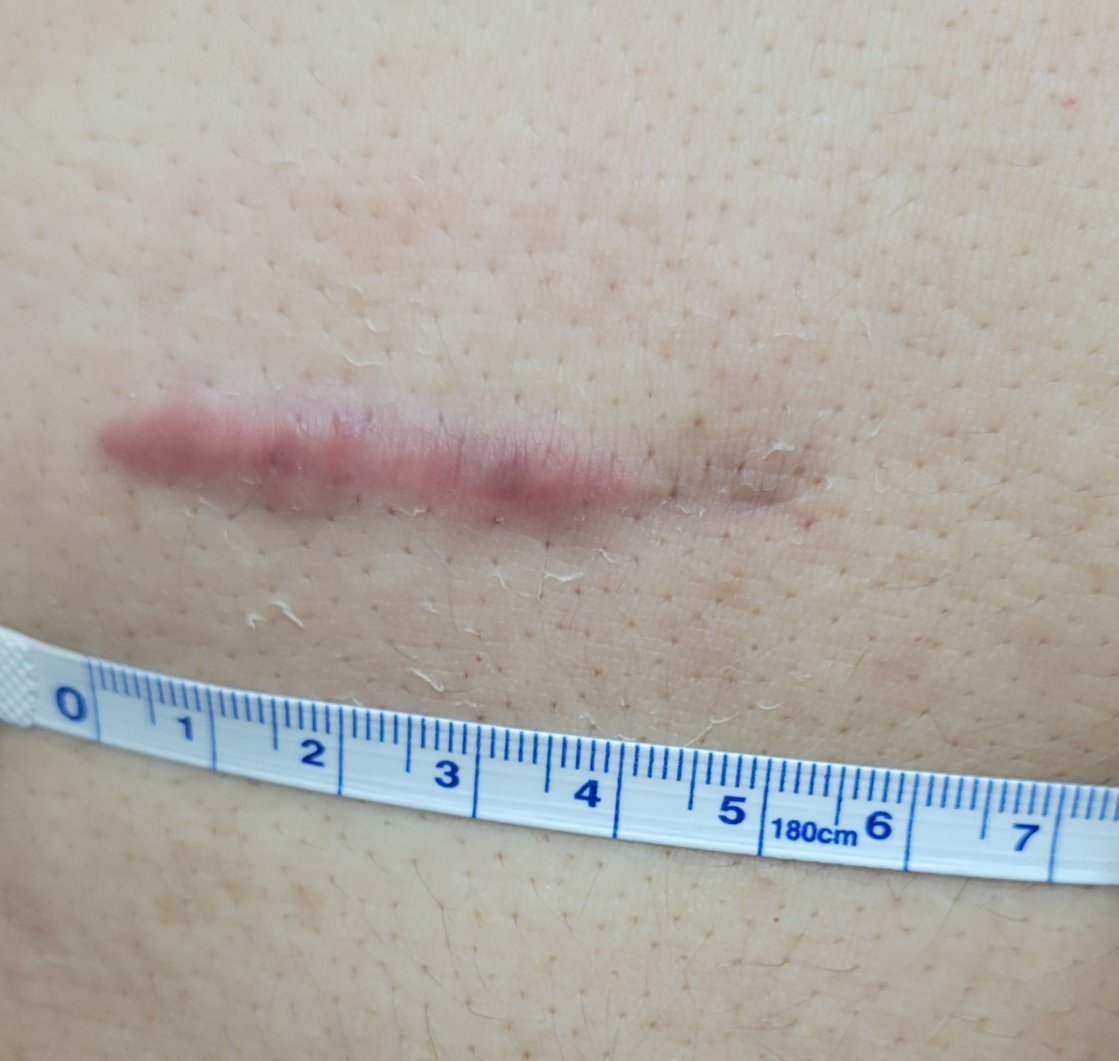
Clinical photograph of the epigastric incision site demonstrating a raised, firm, erythematous scar with localized thickening and irregular surface texture measuring approximately 4 cm at postoperative follow-up The scar corresponds to the prior laparoscopic port site and remains symptomatic with tenderness and pruritus.

At postoperative follow-up, the patient reported partial improvement in pressure and bulging but persistent localized scar pain, irritation, and redness. He denied drainage, fever, or features suggestive of recurrent hernia. The discomfort was described as a burning and pulling sensation, worsened by touch. On examination, the epigastric incision measured approximately 4 cm and appeared raised, firm, and erythematous with localized tenderness (Figure 3). The patient reported partial improvement in irritation and pruritus with the use of Contractubex^®^ gel (heparin, allantoin, and onion extract), applied twice daily.

On physical examination, the epigastric incision appeared elevated, firm, erythematous, and mildly tender, without fluctuance, warmth, drainage, or palpable hernia recurrence. The abdomen was soft and non-distended.

**Figure 4:**
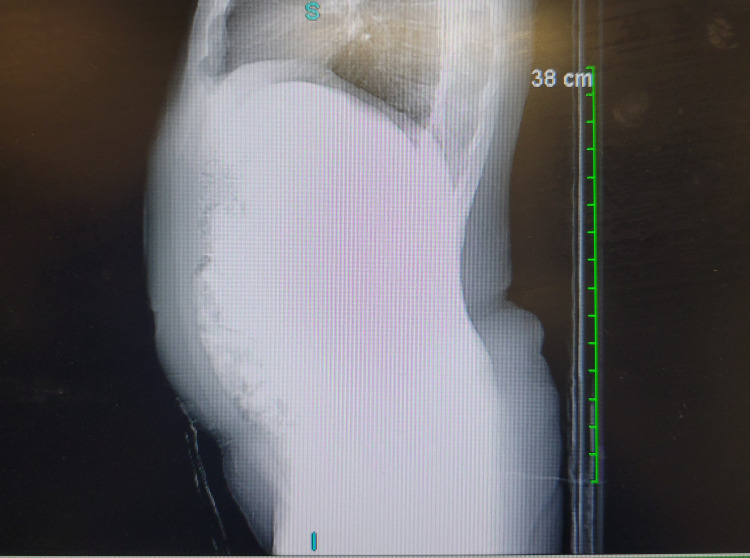
Sagittal CT image of the upper abdomen (non-contrast) obtained on June 27, 2025, demonstrating an intact fascial repair without evidence of recurrent hernia, seroma, abscess, or postoperative fluid collection Partially visualized bowel loops are not dilated. No acute intra-abdominal pathology is identified.

A postoperative CT of the abdomen without contrast, performed on June 27, 2025, demonstrated an unremarkable abdomen, with no free air, fluid collection, lymphadenopathy, or bowel obstruction. The liver, spleen, pancreas, adrenals, kidneys, and aorta were unremarkable. No seroma, abscess, or recurrent hernia was identified. These findings confirmed stable postoperative anatomy and an intact fascial repair (Figure 4).

Given the chronicity of symptoms, postoperative imaging findings, and confirmed histopathology, the patient was diagnosed with recurrent symptomatic hypertrophic scar following open incisional hernia repair. He was advised to continue conservative topical scar therapy, with consideration of serial intralesional corticosteroid injections, silicone sheeting, and dermatologic referral for adjunctive therapies.

## Discussion

Hypertrophic scars are characterized by excessive collagen deposition during wound repair and remain confined to the original wound boundaries, distinguishing them from keloid scars, which extend beyond the wound margins. Their pathogenesis involves abnormal, dysregulated fibroblast activity, prolonged inflammation, and excessive production of the extracellular matrix. These processes are often triggered by factors such as wound depth, delayed healing, location, mechanical tension, infection, and genetic predisposition [5,6]. In this patient, abdominal wound tension following laparoscopic cholecystectomy, combined with a documented postoperative superficial infection, created an environment conducive to hypertrophic scar formation and subsequent incisional hernia development.

Incisional hernia is a recognized complication after abdominal surgery due to disruption of the fascia, with risk factors including advanced age, obesity, diabetes, SSI, and trocar size. SSIs significantly increase the risk of hernia development, as confirmed by meta-analyses, because they impair collagen remodeling and increase fascial dehiscence. Although most trocar-related hernias occur at the umbilical port site, this patient’s postoperative infection and delayed healing became the locus of hernia formation, likely due to a combination of tissue tension, repeated inflammation, and scarring [6,7].

Management of hypertrophic scars is multimodal due to their high recurrence rate after treatment. First-line therapies include tension reduction, silicone gel sheeting, and intralesional corticosteroid injections. Triamcinolone acetonide (TAC) demonstrates the highest efficacy among corticosteroids for scar reduction [4,8]. Corticosteroid injections reduce hypertrophic scar formation by downregulating pro-inflammatory cytokines and growth factors, such as TGF-β, which decreases fibroblast activity and extracellular matrix deposition. These injections ultimately reduce scar thickness and volume, as well as pruritus and pain [4,8]. Limiting factors for corticosteroid treatment include variable efficacy, a high recurrence rate, and steroid resistance, often requiring multiple dermatology clinic visits, which can be challenging for patients in resource-limited settings [8]. Pressure therapy and topical agents may provide symptomatic relief, but evidence for their effectiveness is variable.

Surgical revision is reserved for refractory cases and should be combined with adjuvant therapies to minimize recurrence risk. Evidence shows that the lowest recurrence rates are achieved with multimodal therapy combining surgery plus radiation and/or TAC [7]. This approach targets multiple aspects of hypertrophic scar pathophysiology: surgery removes the bulk of the scar, while radiation and TAC suppress fibroblast proliferation and collagen synthesis, preventing abnormal wound healing and reducing recurrence.

The patient’s allergic reaction to cephalexin, a commonly prescribed beta-lactam antibiotic for skin and soft-tissue infections, is consistent with known IgE-mediated hypersensitivity and necessitated immediate discontinuation [9]. Symptomatic improvement with topical Contractubex^®^, a formulation containing heparin, allantoin, and onion extract, aligns with experimental evidence showing improved wound architecture and reduced scar thickness in animal models [4].

This case suggests a multifactorial contribution to hypertrophic scar recurrence, including postoperative infection, delayed wound healing, mechanical tension, and prior scar pathology. These factors interact to produce persistent symptoms. Evidence-based management requires early intervention, multimodal therapy, and individualized follow-up to optimize outcomes [4-6,7]. However, interpretation of these findings should consider the report’s limitations, including its single-patient design, which restricts generalizability and precludes causal inference, and the relatively limited duration of postoperative follow-up, which limits assessment of long-term recurrence risk and durability of treatment outcomes.

## Conclusions

This case illustrates the potential contributions of postoperative infection, delayed wound healing, and mechanical stress to the development of hypertrophic scarring and subsequent incisional hernia formation. The patient’s clinical course highlights the challenges of managing postoperative hypertrophic scars, particularly when infectious complications exacerbate fibroproliferative responses and compromise fascial integrity. Conservative measures, including topical scar therapy (e.g., Contractubex^®^) and tension reduction, remain foundational components of a multimodal treatment approach. However, adjunctive interventions such as intralesional corticosteroid injections, laser therapy, or combination modalities may be required for sustained symptom control in more severe or recurrent cases. Although limited by the inherent constraints of a single case, this report emphasizes the importance of early recognition of postoperative wound complications and timely implementation of multimodal scar management strategies to mitigate recurrence and potentially reduce postoperative complications.
